# An Approach to Anesthetic Management of Venous Anomalies and Outflow Reconstruction in Liver Transplants

**DOI:** 10.7759/cureus.44059

**Published:** 2023-08-24

**Authors:** Hamza Malick, Sabine Itani, Daniel C Gunn, Amar Gupta, Giuliano Testa, Saravanan Ramamoorthy

**Affiliations:** 1 College of Medicine, Texas A&M College of Medicine, Dallas, USA; 2 College of Medicine, Texas A&M College of Medicine, Bryan, USA; 3 Department of Anesthesiology, Baylor University Medical Center, Dallas, USA; 4 Department of Transplant Surgery, Baylor University Medical Center, Dallas, USA; 5 Anesthesiology and Perioperative Medicine, US Anesthesia Partners, Dallas, USA

**Keywords:** venous out-flow, liver transplant anesthesia, ischemia-reperfusion injury, hepatic congestion, living donor liver transplant-ldlt

## Abstract

Hepatic venous outflow is a pivotal factor in liver transplant. However, venous anomalies and the potential for hepatic venous congestion continue to remain major points of concern to ensure the viability of transplanted livers and maximize regenerative capacity. We present a 66-year-old patient undergoing liver transplantation who was found to have anomalous venous drainage requiring venous anastomoses. To ensure adequate venous flow and minimize the possibility of graft congestion and liver dysfunction, the anesthetic management of the patient's hemodynamic status was of utmost importance. The use of osmotic diuretics and intraoperative sonography was used to ensure adequate perfusion.

## Introduction

Successful hepatic venous outflow is universally accepted as a pivotal factor in the success of liver transplantation. However, venous anomalies and the potential for hepatic venous congestion continue to be major concerns for ensuring the viability of transplanted livers and maximizing regenerative capacity. The need for adequate perfusion while mitigating possible venous congestion in complicated outflow anomalies has been established in the literature previously. In this case, we discuss a plan for anesthetic management and intraoperative care used to mitigate such complications. The use of diuretics like mannitol and unique surgical procedures that included anastomosing adjacent recurring outflow tracts into single conduits are discussed. The patient provided written consent to publish this case with appropriate Health Insurance Portability and Accountability Act authorization.

## Case presentation

**Figure 1:**
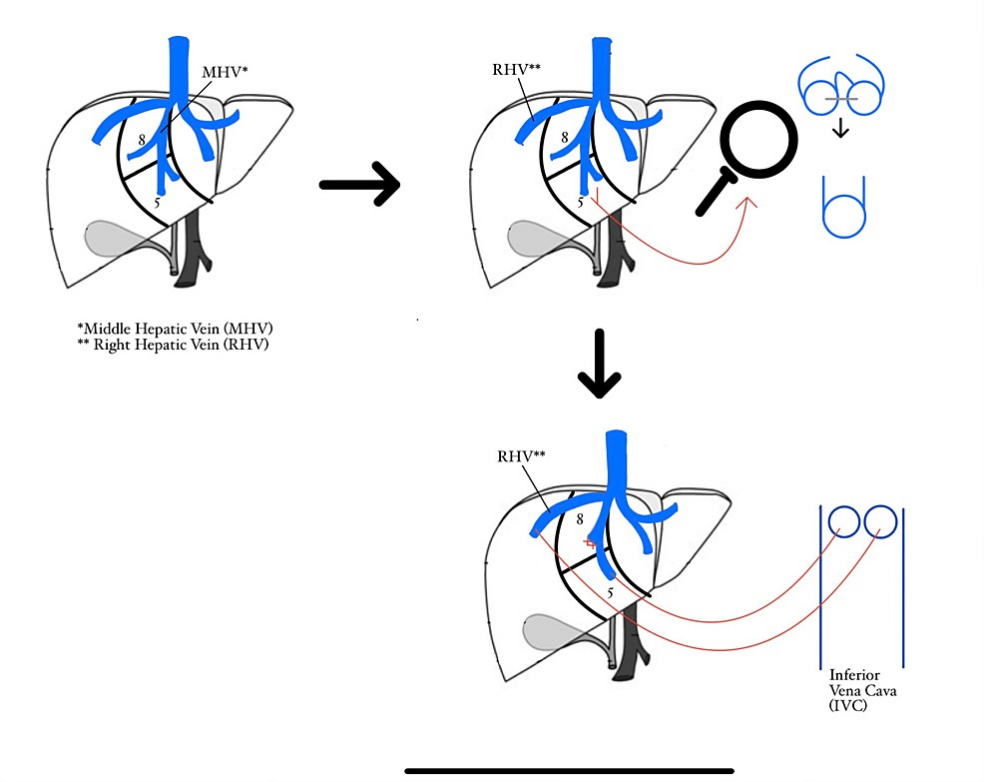
Back Table Anastomosis of the Venous Anomalies Present in the Middle Hepatic Vein

A 66-year-old patient underwent a living donor liver transplant due to end-stage liver failure secondary to cirrhosis due to autoimmune hepatitis. During the donor liver hepatectomy, significant anomalous venous drainage was identified, requiring a back table anastomosis of venous anomalies branching from the middle hepatic vein of the donor liver (Figure 1). Additionally, significant outflow from adjacent veins in segment 5 of the liver was observed and eventually joined into a single orifice (Figure 1).

**Figure 2:**
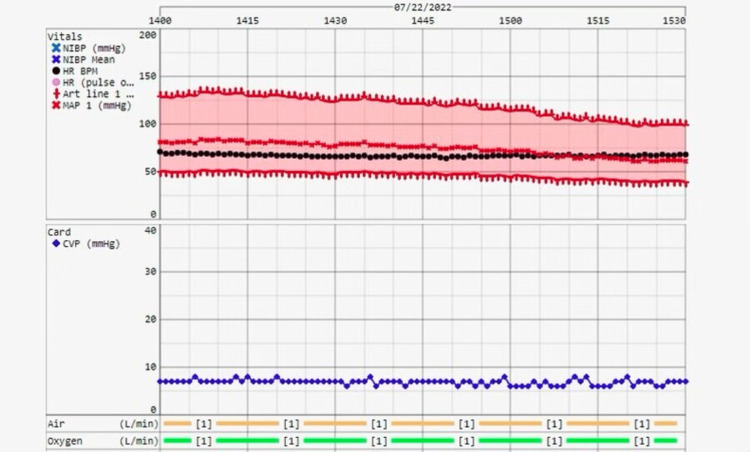
CVP was maintained at 8mmHg throughout the surgery CVP: Central venous pressure

In the operating room (OR), general endotracheal anesthesia was induced and the patient was successfully intubated. The patient's pre-operative blood pressure was 150/63. All other pre-operative vitals and hemodynamic status were reported as stable. Invasive blood pressure monitoring was established through a left brachial arterial line, preferenced by institution-specific guidelines for liver transplant due to minimizing effects on blood pressure from peripheral vasoplegic states. A double-lumen catheter was placed into the internal jugular vein for central venous pressure (CVP) monitoring. A transesophageal echocardiography (TEE) probe was inserted for dynamic intraoperative volume assessment. The baseline CVP was measured at 12 mmHg, and TEE showed a euvolemic left ventricle with mild tricuspid regurgitation. To maintain appropriate diuresis a low CVP was desired to mitigate possible hepatic congestion following reperfusion. A mannitol infusion (25%) at 1g/kg was initiated at a rate of 100 mL per hour before incision. Additionally, 40 mg of furosemide was administered after reperfusion to maintain a CVP of less than 8 mmHg (Figure 2).

After successful attachment of the right portal vein of the graft with the main portal vein of the recipient, an anastomosis of the left and middle hepatic veins was required due to observed discoloration of segments 5 and 8 of the donor liver. Adequate graft reperfusion was achieved shortly after creating the new conduit. Intraoperative sonography was used to confirm adequate perfusion after all appropriate donor-recipient outflow conduits were anastomosed and after reperfusion of the transplanted liver. Throughout the 11-hour procedure, the patient received a total of 500 mL of 25% mannitol, along with 1,000 mL of lactated ringers, 1,000 mL of 5% albumin, 300 mL of fresh frozen plasma (FFP), 200 mL of platelets, and 500 mL of packed red blood cells (PRBC). The estimated blood loss was 1,000mL and the estimated urine output was 1,500 mL. The patient remained hemodynamically stable without the need for vasoactive anesthetics and was successfully extubated. Adequate postoperative pain management was achieved through scheduled tramadol and acetaminophen with codeine. Subsequently, the patient was transferred to the intensive care unit (ICU) for appropriate post-operative recovery. On post-operative day 1, the patient was discharged from the ICU, and on post-operative day 5, the patient was discharged home without any complications.

## Discussion

**Figure 3:**
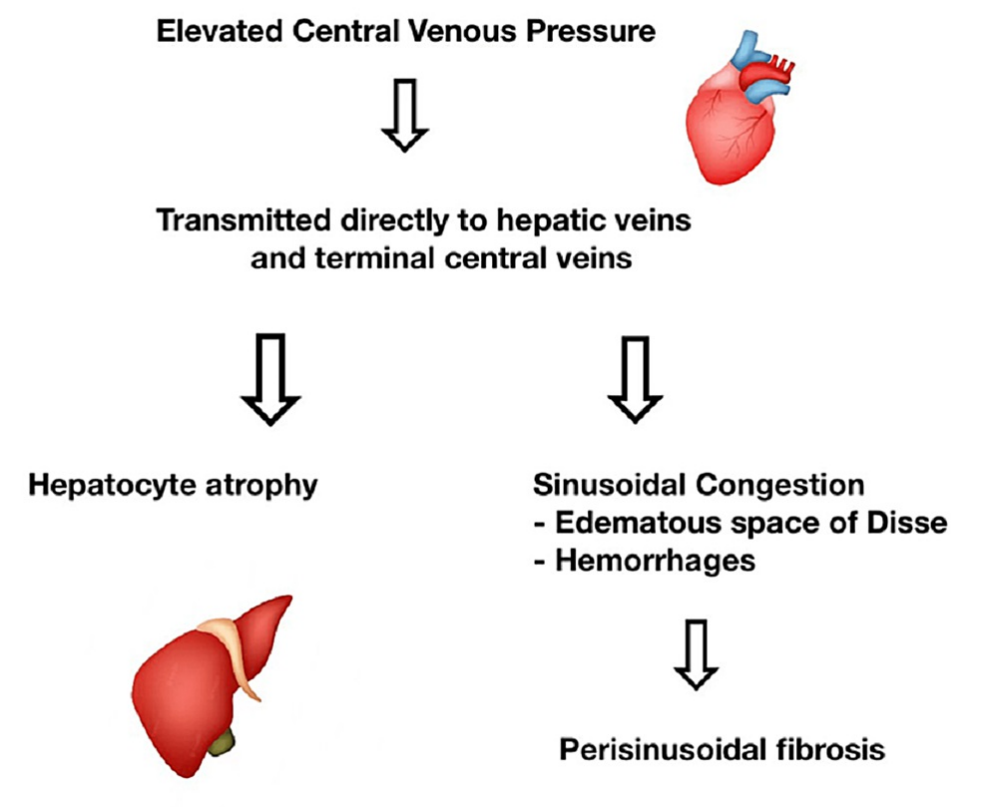
Intra-operative control of lower CVP levels is necessary to avoid hepatic congestion and promote organ survival CVP: Central venous pressure

Liver transplantation has been widely praised as a cure for patients with acute and chronic end-stage liver failure [1]. As the mortality and morbidity associated with hepatic dysfunction remain prevalent, the need for liver donors continues to rise, and advancements in clinical and surgical practices are necessary to provide the best possible care and survival for the transplanted liver. One potential complication of liver transplants is hepatic congestion and hypoperfusion of liver tissue. Adequate perfusion of grafted tissue and targeted intra-operative control of lower CVP levels is crucial for the survival of donor livers in the recipient (Figure 3). While the hepatic arteries are often managed through total arterial circulation, they only account for 25% of total hepatic blood flow [2]. The majority of hepatic blood flow and perfusion depends on hepatic and portal venous flow which lack autoregulatory buffers and thus require optimal perfusion pressure and venous outflow [2].

Previous studies have emphasized the importance of vascular outflow tracts and the reconstruction of their unique anatomical anomalies for the survival and perfusion of grafted liver tissue [3]. The necessity for venoplasty and the formation of anastomosed conduits from multiple small hepatic vein tributaries has been identified as an actionable requirement during surgery to mitigate the risk of hypoperfusion and hepatic congestion [3,4]. Similarly, studies have shown the efficacy of controlled intra-operative CVP in mitigating hepatic congestion and promoting liver viability. However, larger randomized control trials are still needed to further establish these findings. The presented case describes an anesthetic practice that utilized osmotic diuretics like mannitol and loop diuretics like Lasix to maintain adequate CVP control. Intraoperative transesophageal echocardiography (TEE) was also used to monitor intraoperative volume status, as it is well-established in practice [5]. Intraoperative sonography was employed to ensure adequate perfusion of liver segments after donor-recipient outflow conduits were anastomosed. These approaches were based on prior studies that successfully used mannitol to attenuate post-reperfusion syndrome in liver transplants with prolonged ischemic times [6]. Similarly, intraoperative sonography was utilized to identify early signs of tissue failure and hepatic venous congestion, which are commonly observed in the early post-transplant period and are significant causes of early graft failure in transplanted livers [4].

Through the measures listed above, the recipient successfully maintained the viability of the transplanted organ, minimizing the risk of hepatic congestion and post-reperfusion syndrome. The patient continued to do well and was discharged without any significant complications. This case report may function to further the understanding of intraoperative management and anesthetic principles of diuretics and sonography in solid organ transplants, particularly in living donor transplants with anatomical anomalies. Further studies on these principles of care remain necessary.

## Conclusions

The presented case highlights the importance of addressing venous anomalies and hepatic venous outflow in liver transplantation to ensure the viability and success of the transplanted liver. Hepatic venous congestion remains a significant concern, and the adoption of unique anesthetic management and intra-operative care can play a crucial role in mitigating potential complications. The use of intra-operative diuretics to keep CVP low, combined with the application of TEE and sonography, aided in preserving organ viability and reducing the risk of venous congestion.

As the demand for liver donors continues to rise, advancing anesthetic management and surgical practices to optimize liver transplantation outcomes is essential. This case serves as a valuable contribution to the growing body of knowledge on potential strategies for anesthetic principles and their unique application in liver transplantation. Further research and larger-scale controlled trials may be necessary.
